# Room for improvement? A survey of the methods used in systematic reviews of adverse effects

**DOI:** 10.1186/1471-2288-6-3

**Published:** 2006-01-27

**Authors:** Su Golder, Yoon Loke, Heather M McIntosh

**Affiliations:** 1 Centre for Reviews and Dissemination (CRD), University of York, York, YO10 5DD, UK; 2 University of East Anglia Norwich NR4 7TJ, UK; 3 NHS Quality Improvement Scotland, Delta House, 50 West Nile St, Glasgow, G1 2NP, UK

## Abstract

**Background:**

Although the methods for conducting systematic reviews of efficacy are well established, there is much less guidance on how systematic reviews of adverse effects should be performed.

**Methods:**

In order to determine where methodological research is most needed to improve systematic reviews of adverse effects of health care interventions, we conducted a descriptive analysis of systematic reviews published between 1994 and 2005. We searched the Database of Abstracts of Reviews of Effects (DARE) and The Cochrane Database of Systematic Reviews (CDSR) to identify systematic reviews in which the primary outcome was an adverse effect or effects. We then extracted data on many of the elements of the systematic review process including: types of interventions studied, adverse effects of interest, resources searched, search strategies, data sources included in reviews, quality assessment of primary data, nature of the data analysis, and source of funding.

**Results:**

256 reviews were included in our analysis, of which the majority evaluated drug interventions and pre-specified the adverse effect or effects of interest. A median of 3 resources were searched for each review and very few reviews (13/256) provided sufficient information to reproduce their search strategies. Although more than three quarters (185/243) of the reviews sought to include data from sources other than randomised controlled trials, fewer than half (106/256) assessed the quality of the studies that were included. Data were pooled quantitatively in most of the reviews (165/256) but heterogeneity was not always considered. Less than half (123/256) of the reviews reported on the source of funding.

**Conclusion:**

There is an obvious need to improve the methodology and reporting of systematic reviews of adverse effects. The methodology around identification and quality assessment of primary data is the main concern.

## Background

While the assessment of adverse effects in systematic reviews of health care interventions is undoubtedly essential, there are significant methodological challenges in undertaking such reviews [1,2]. Some guidance is available from The Cochrane Collaboration [3] but the lack of empirical knowledge remains a major handicap to reviewers. The difficulty of sensitive searching in MEDLINE and EMBASE has already been identified [4-6], and there is still considerable uncertainty over the type and quality of studies that should be included in these systematic reviews. A closer look at the content and methods of published reviews is needed to identify priority areas for methodological research.

Our aim was to describe the general characteristics of systematic reviews published from 1994 to 2005 that focussed primarily on adverse outcomes associated with healthcare interventions, in order to identify priority areas for methodological research. The decision to begin by looking at systematic reviews with adverse effects as the primary outcome was a pragmatic one.

## Methods

We searched The Cochrane Database of Systematic Reviews (CDSR) and the Database of Abstracts of Reviews of Effects (DARE) via The Cochrane Library, plus DARE via the Centre for Reviews and Dissemination (CRD) website [see Additional file 1]. These databases were chosen because they are major, comprehensive collections of systematic reviews. For instance, DARE is compiled through rigorous monthly searches of bibliographic databases (including MEDLINE and EMBASE) as well as handsearching of key journals, grey literature and regular searches of the internet. We did not place any language restrictions on the searches and we aimed to retrieve systematic reviews published from 1994 onwards.

Two researchers independently screened titles and abstracts and selected full papers for inclusion. Any discrepancies between the researchers were resolved by discussion and consensus. A review was included if the primary aim was to evaluate adverse effects, known to be, or suspected to be, associated with an intervention. This was regardless of whether the review's hypothesis stated that the intervention increased or reduced the outcome. Papers that investigated the complete safety profile of an intervention were included if this was their primary aim.

We abstracted pre-defined descriptive data using a standardised form designed for this study. For each review, we collected baseline data on the types of intervention reviewed, scope of the adverse effects evaluation, and the source of funding. We then looked at specific methodological issues which were raised in the Cochrane Handbook guidance. This covered the topics of search strategy, resources searched, types of studies reviewed, quality assessment, and data analysis.

## Results

**Figure 1:**
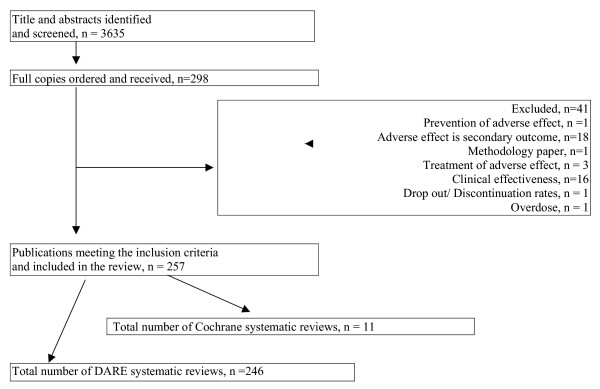
Summary of systematic review identification, retrieval and inclusion/exclusion.

**Figure 2:**
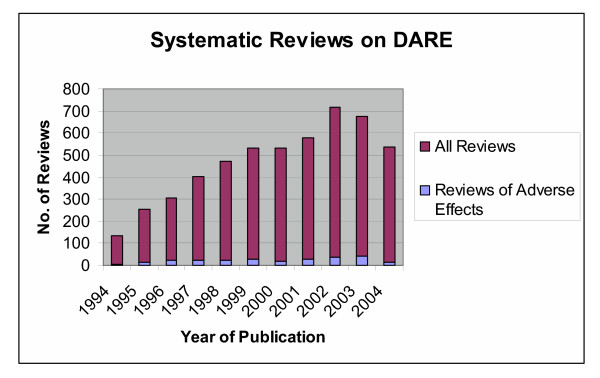
***Proportion of systematic reviews of adverse effects relative to all reviews on DARE by year of publication***. The results shown for 2004, and to a lesser extent 2003, are low because some records are still in the DARE production process.

From 3635 titles and abstracts screened, 298 full reports were retrieved and 256 reviews met our inclusion criteria [see Figure 1]. Most of the included reviews were identified from DARE with only 11 Cochrane reviews identified from CDSR. The number of reviews focusing on adverse effects is increasing over time but the proportion of these reviews relative to all reviews on DARE has remained between 2% and 8% [see Figure 2].

### Types of interventions studied

The included reviews are dominated by those evaluating the adverse effects of drugs (64%), with only a few studies looking at surgical procedures (8%) or other physical interventions such as acupuncture (5%) [see Table 1]. The most commonly reviewed drugs were female hormonal agents used either as replacement therapy (24 reviews) or as contraception (14 reviews).

**Table 1:** Number of reviews by type of intervention

**Year of publication**	**Type of intervention**					**Total number of reviews**
		
	**Drug**	**Surgical/dental**	**Physical**	**Diagnostic/screening**	**Other**	
**1994**	5	0	0	0	0	5
**1995**	9	0	0	0	3	12
**1996**	9	6	2	0	5	21
**1997**	15	2	1	0	6	29
**1998**	18	2	0	0	4	23
**1999**	21	1	0	1	4	27
**2000**	13	2	1	0	2	17
**2001**	17	1	2	0	9	27
**2002**	19	7	3	0	11	40
**2003**	26	1	3	0	14	44
**2004**	12	1	1	0	3	17
**Jan-Apr 2005**	0	0	0	0	0	0
**Total**	164	20	13	1	56	256

The sum of reviews by intervention is greater than the total number of reviews because 5 reviews examined 2 (4 reviews) or 3 (1 review) types of intervention (e.g. drug and non-drug antihypertensive therapy)

### Scope of adverse effects evaluation

Rather than investigating all potential adverse effects, 80% (208/256) of the reviews preferred to focus on pre-specified adverse effects outcomes.

### Sources of funding

Less than half of the reviews (123/256) provided information on the source of funding. Of the reviews that did report financial support 85 were independent sources, 32 could represent a conflict of interest (largely a drug manufacturer), and 6 said they received no financial support.

### Resources searched

Nearly all of the reviews (250/256) listed the resources searched to identify studies. The median number of electronic databases searched was only 2 (range 0 to 25), MEDLINE being the most popular (242/256) followed by EMBASE (90/256). Many reviews (218/256) reported searching at least one additional source with the references from included studies being by far the most popular (199/256), followed by contacting experts in the field (58/256).

### Search strategies

The authors reported their search strategies in three quarters (196/256) of the reviews, but very few (13/256) provided sufficient information to reproduce the search. Almost half of the reproducible searches were conducted by a qualified information specialist (6/13) compared to only 7% (17/243) of those that did not report enough detail. In most cases (165/256) it was unclear whether the searches had been restricted by language; only 15% (38/256) explicitly restricted by language and 21% (53/256) explicitly did not.

Even when search strategies were reported they were invariably limited. Many did not use any synonyms (149/196) or truncation (171/196). Of those that indicated which fields were searched almost half (20/43) relied solely on indexing or solely on text words.

### Data sources included in reviews

About 5% (13/256) of reviews did not report on the types of studies included in their analysis. While 63% (154/243) of the remaining reviews sought to include studies that compared the intervention with a control, only 28% (68/243) of reviews limited themselves to data from randomised controlled trials (RCTs). Cohort studies (75/243) and case-control studies (67/243) were included in about one third of the reviews whereas case series (21/243) and case reports (28/243) were included in approximately 10%.

### Quality assessment of primary data

Less than half (106/256) of the reviews specified assessment of the quality of the included studies in the methods section, or alluded to it by describing in the methods data extraction that included indicators of study quality. Forty-nine of the 106 reviews used existing quality assessment instruments, mostly to assess RCTs. Although some of the reviews evaluated data from a variety of study designs, evidence hierarchies were rarely used (12/256).

### Nature of the data analysis

Meta-analysis was used to pool data in over 60% (165/256) of the reviews and almost 90% (145/165) of those reviews assessed heterogeneity. In 10% (27/256) of the reviews, overall event rates were derived by simply adding up the numbers of events reported in each of the included studies and only 37% (10/27) of those reviews considered heterogeneity. Sixty-four reviews presented only narrative synthesis; 20/64 gave reasons for not pooling data quantitatively. Heterogeneity was the most common reason given (12/20).

## Discussion

Our interest lies in the methods of addressing adverse effects in systematic reviews. Our finding that this type of review accounts for approximately 4% of systematic reviews is consistent with an earlier survey of reviews on The Cochrane Library and MEDLINE [7]. There are several findings regarding the nature and methodology of the systematic reviews of adverse effects that merit further discussion.

### Types of interventions studied

One area of concern is that systematic reviews identified in this study have mainly been directed towards the adverse effects of pharmacological interventions. This emphasis on drug therapy needs to be redressed, given that surgical and other physical interventions are widely used in healthcare, and may have equally important or serious adverse effects.

### Scope of adverse effects evaluation

Many of the reviews also tended to be directed towards evaluating pre-specified adverse outcomes of interest, which indicates that the reviewers usually had an *a priori *hypothesis when conducting the review and that the detection of new unrecognised adverse effects was of lesser interest. It may also reflect the relative ease of concentrating on a few major outcomes rather than spreading oneself too wide. Indeed this mirrors the experience of researchers who have argued that the focused approach is better able to yield clinically relevant results [8] than broad, unfocused reviews.

### Sources of funding

Under reporting of the source of funding is evident from our findings. Source of funding is obviously important as it may be in the manufacturer's interest to highlight the positive safety aspects of their product. Missing data on funding meant we could not conduct subgroup analysis with confidence to investigate whether the conclusions of manufacturer-sponsored reviews differed from independently or non-sponsored studies. Other researchers have found that trials funded by drug companies are more likely to have outcomes that favour the sponsor's product [9]. It is possible that a similar bias exists in some systematic reviews of adverse effects, where the review may downplay any safety concerns. Here, we urge systematic reviewers to disclose their source of funding and any competing interests. We also recommend further research into how the analysis and reporting of adverse effects may or may not vary with the source of funding.

### Resources searched

The number of sources searched was low and even lower than reported in a similar study of systematic reviews of qualitative data [Booth A. ISSG Meeting 2005]. Not surprisingly, as with reviews of efficacy, nearly all the reviews here searched MEDLINE. This is most likely due to accessibility and popularity rather than the usefulness of MEDLINE as a source of information on adverse effects. For example, EMBASE is likely to be a better source of information on drug-related adverse effects than MEDLINE [5]. We recommend that reviewers adopt more comprehensive searches across a wider range of sources. Researchers who are not familiar with the differing content of databases may end up relying too heavily on MEDLINE, and there is a need for research and guidance into prioritizing the most useful databases when searching for information on adverse effects.

### Search strategies

The set of reviews described here provided only limited information on the techniques they employed to identify primary studies and the search strategies that were reported tended to be of poor quality. Synonyms, truncation and the use of text words in combination with indexing were rarely used, yet these are all crucial aspects of a sensitive search strategy as required by systematic reviews. We believe that search strategies should be reported more clearly and that it may be more appropriate to use broader search strategies when searching for studies for a systematic review.

### Data sources included in reviews

It was interesting to find that substantial numbers of reviews chose to extend their analysis beyond data from controlled trials, and into the realms of observational studies. This probably stems from the widely held view that short-term trials in selected populations are not the best study design for the evaluation of rare or long-term adverse effects, and that reviewers may find it necessary to utilize other data sources.

### Quality assessment of primary data

The areas of quality assessment and data analysis are of some concern here. It is difficult to judge the reliability of the primary data, given that less than half the reviews reported any attempt to assess quality. This may be explained by the absence of a defined quality assessment tool, especially with regards to observational studies. Moreover, the evidence hierarchies that are often used in the evaluation of clinical efficacy may not be appropriate for adverse effects information, and there is a need to develop appropriate ways of assessing quality of adverse effects data from different types of studies.

### Nature of the data analysis

It is clear from our data that there are many occasions where meta-analysis is not the analytic method of choice, and this may reflect the problems with handling diverse sources of data. On the other hand, some reviewers who pool data appear to disregard differences between the studies. There is a clear need for further research and guidance into the appropriate methods of synthesizing data that arise from a wide range of study designs.

### Limitations of our analysis

DARE and CDSR were awkward to search for the type of reviews we were interested in and it is possible that we missed some relevant reviews. It is not possible to search using floating subheadings in DARE via the CRD interface, or to limit the search to the 'outcomes' field in DARE via The Cochrane Library interface, or to limit the search to the 'objectives' section in Cochrane review abstracts on CDSR. Our sample of reviews will also have been influenced by changes in the production of DARE over time, including more sensitive searches to identify systematic reviews and tighter criteria for inclusion of reviews on the database. Only 5/256 reviews in our sample were non-English language publications, which may not be a fair representation because CDSR only has English language reviews and translation capacity delays some foreign language reviews reaching DARE.

Although this systematic review was limited to reviews in which adverse effects were a primary outcome, much of the methodology should be relevant to systematic reviews in which adverse effects are secondary outcomes. Such reviews will often require additional searches to those for the clinical effects, which are often limited to RCTs. Similarly, there are issues surrounding quality assessment and the pooling of data from different data sources. It would be very useful to survey the methods of assessing adverse effects data in systematic reviews where adverse effects have been considered to be a secondary aim, and to compare those findings against ours.

## Conclusion

Two areas of methodology that should be prioritised for improvement are searching and quality assessment. Although questions on the optimal search strategies remain to be answered, the basic quality of searching could easily be improved by more involvement from an information specialist. Future research could be undertaken to investigate how the deficiencies in the searches identified here could alter the conclusions of a review. This could be done by case studies in which the yield and consequent findings from more comprehensive searches are compared to basic searching.

The lack of a defined and empirically tested quality assessment tool is a major problem. In order to be able to form reliable conclusions, readers of systematic reviews need to have some indication of the reliability and validity of the primary adverse effects data. The development of this quality instrument could be mirrored on the model successfully used in producing the tool for the quality assessment of studies of diagnostic accuracy [10].

There is also undoubtedly a need to improve the quality of reporting. Incomplete reporting might partly be due to limited space in journal articles, but space is often wasted on vague or ambiguous reporting that is particularly noticeable in the reporting of search strategies. It is important that guidelines on the reporting of systematic reviews continue to incorporate evidence of actual reporting deficiencies from published reviews of adverse effects [11,12].

## Competing interests

The author(s) declare that they have no competing interests.

## Authors' contributions

SG participated in the conception and design of the study, carried out the searches, scanned search results, carried out data extraction, and helped draft the manuscript. YL carried out data extraction, and helped draft the manuscript. HM conceived the study, participated in the design of the study, scanned search results, carried out data extraction, and helped draft the manuscript. All authors read and approved the final manuscript.

## Pre-publication history

The pre-publication history for this paper can be accessed here:



## Supplementary Material

Additional File 1 Search strategies to retrieve systematic reviews of adverse effects in DARE and CDSR Click here for file
