# Supplementary material for: Room for improvement? A survey of the methods used in systematic reviews of adverse effects
Source: BMC Med Res Methodol. 2006 Jan 27;6:3. doi: 10.1186/1471-2288-6-3 (PMC1402311; doi:10.1186/1471-2288-6-3)
Supplement: Additional File 1 — Search strategies to retrieve systematic reviews of adverse effects in DARE and CDSR [file 1471-2288-6-3-S1.doc]

**Search strategies to retrieve systematic reviews of adverse effects in DARE and CDSR**

**Database of Abstracts of Reviews of Effects (DARE) via CRD**

**Searched: 31/03/05**

(safe or safety or adverse or tolerability or toxicity or toxic or adrs or adr or tolerance or tolerate or harm or harms or harmful or complication$ or risk or risks)/xoa OR side(w)effect$/xoa OR undesirable(w)effect$/xoa

treatment(w)emergent/xoa

(safe or safety or adverse or tolerability or toxicity or toxic or adrs or adr or tolerance or tolerate or harm or harms or harmful or complication$ or risk or risks)/ttl OR side(w)effect$/ttl OR undesirable(w)effect$/ttl

treatment(w)emergent/ttl

**Database of Abstracts of Reviews of Effects (DARE) via Cochrane Library**

**Searched: 31/03/05**

#1 Any MeSH descriptor with qualifier: AE in MeSH products

#2 Any MeSH descriptor with qualifier: DE in MeSH products

#3 Any MeSH descriptor with qualifier: CO in MeSH products

#4 Any MeSH descriptor with qualifier: PO in MeSH products

#5 Any MeSH descriptor with qualifier: TO in MeSH products

#6 Any MeSH descriptor with qualifier: CI in MeSH products

#7 MeSH descriptor Drug Hypersensitivity explode all trees in MeSH products

#8 MeSH descriptor Drug Toxicity explode all trees in MeSH products

#9 MeSH descriptor Product Surveillance, Postmarketing explode all trees in MeSH products

#10 #1 or #2 or #3 or #4 or #5 or #6 or #7 or #8 or #9

**Cochrane Database of Systematic Reviews (CDSR) via Cochrane Library**

**Issue 1 2005**

**Searched: 31/03/05**

This database uses automatic pluralisation, so terms “harms and risks” are not required as the terms “harm and risk” are already in the strategy.

#1 Any MeSH descriptor with qualifier: AE in MeSH products

#2 Any MeSH descriptor with qualifier: DE in MeSH products

#3 Any MeSH descriptor with qualifier: CO in MeSH products

#4 Any MeSH descriptor with qualifier: PO in MeSH products

#5 Any MeSH descriptor with qualifier: TO in MeSH products

#6 Any MeSH descriptor with qualifier: CI in MeSH products

#7 MeSH descriptor Drug Hypersensitivity explode all trees in MeSH products

#8 MeSH descriptor Drug Toxicity explode all trees in MeSH products

#9 MeSH descriptor Product Surveillance, Postmarketing explode all trees in MeSH products

#10 (safe or safety or adverse or tolerability or toxicity or toxic or adrs or adr or tolerance or tolerate or harm or harms or harmful or complication* or risk or risks) near/20 objective* in Abstract in all products

#11 (side next effect*) near/20 objective* in Abstract in all products

#12 (undesirable next effect*) near/20 objective* in Abstract in all products

#13 (treatment next emergent) near/20 objective* in Abstract in all products

#14 (safe or safety or adverse or tolerability or toxicity or toxic or adrs or adr or tolerance or tolerate or harm or harms or harmful or complication* or risk or risks) in Record Title in all products

#15 (side next effect*) in Record Title in all products

#16 (undesirable next effect*) in Record Title in all products

#17 (treatment next emergent) in Record Title in all products

#18 #1 or #2 or #3 or #4 or #5 or #6 or #7 or #8 or #9 or #10 or #11 or #12 or #13 or #14 or #15 or #16 or #17 or #18
